# Developing standards for malaria microscopy: external competency assessment for malaria microscopists in the Asia-Pacific

**DOI:** 10.1186/1475-2875-11-352

**Published:** 2012-10-24

**Authors:** Sania Ashraf, Angie Kao, Cecilia Hugo, Eva M Christophel, Bayo Fatunmbi, Jennifer Luchavez, Ken Lilley, David Bell

**Affiliations:** 1 Rollins School of Public Health, Emory University, Georgia, USA; 2 School of Nursing and Health Studies, Georgetown University, Washington, USA; 3 Asian Collaborative Training Network for Malaria (ACTMalaria), Manila, Philippines; 4 WHO Regional Office for the Western Pacific, Manila, Philippines; 5 Research Institute for Tropical Medicine (WHO Collaborating Centre), Alabang, Philippines; 6 Australian Army Malaria Institute (WHO Collaborating Centre), Brisbane, Australia; 7 Foundation for Innovative New Diagnostics (FIND), Geneva, Switzerland

**Keywords:** Malaria microscopy, Diagnostics, Quality assurance programmes

## Abstract

**Background:**

Malaria diagnosis has received renewed interest in recent years, associated with the increasing accessibility of accurate diagnosis through the introduction of rapid diagnostic tests and new World Health Organization guidelines recommending parasite-based diagnosis prior to anti-malarial therapy. However, light microscopy, established over 100 years ago and frequently considered the reference standard for clinical diagnosis, has been neglected in control programmes and in the malaria literature and evidence suggests field standards are commonly poor. Microscopy remains the most accessible method for parasite quantitation, for drug efficacy monitoring, and as a reference of assessing other diagnostic tools. This mismatch between quality and need highlights the importance of the establishment of reliable standards and procedures for assessing and assuring quality. This paper describes the development, function and impact of a multi-country microscopy external quality assurance network set up for this purpose in Asia.

**Methods:**

Surveys were used for key informants and past participants for feedback on the quality assurance programme. Competency scores for each country from 14 participating countries were compiled for analyses using paired sample *t*-tests. In-depth interviews were conducted with key informants including the programme facilitators and national level microscopists.

**Results:**

External assessments and limited retraining through a formalized programme based on a reference slide bank has demonstrated an increase in standards of competence of senior microscopists over a relatively short period of time, at a potentially sustainable cost. The network involved in the programme now exceeds 14 countries in the Asia-Pacific, and the methods are extended to other regions.

**Conclusions:**

While the impact on national programmes varies, it has translated in some instances into a strengthening of national microscopy standards and offers a possibility both for supporting revival of national microcopy programmes, and for the development of globally recognized standards of competency needed both for patient management and field research.

## Background

Although 100 years have elapsed since Ross introduced improvements to malaria diagnosis through Romanowsky-stained, thick blood film microscopy [1], light microscopy continues to be fundamental to good diagnosis. Malaria remains an important disease worldwide, responsible for an estimated 225 million cases of illness and 781,000 deaths globally in 2009, predominantly in Africa [2]. In much of Asia and the Western Pacific current advances in malaria control show positive progress with only 262,474 confirmed cases in the Western Pacific Region in 2010 [2]. Similar declines are seen in South America and parts of Africa. However, clinical diagnosis based solely on symptoms and signs remains common [2]. With this decline in malaria incidence, reliance on the clinical diagnosis of malaria is no longer tenable and an accurate parasite-based confirmation is increasingly important for directing treatment and patient management [3]. Such parasite-based diagnosis must be highly accurate, as negative results are used as a basis for withholding treatment that would otherwise be potentially life-saving in a true case of malaria. Proper diagnosis minimizes overtreatment of populations, which in some instances may increase the potential for selecting strains resistant to anti-malarial drugs.

Accurate diagnosis is achieved either by the microscopic examination of a stained blood film, or by use of an antigen-detecting lateral flow test (rapid diagnostic test – RDT), and both are now accepted as a basis for the management of malaria disease [4]. Light microscopy remains vital to national malaria programmes for the diagnosis and management of severe malaria, routine drug efficacy monitoring and clinical studies, and may be more cost-effective in high throughput settings. Rapid diagnostic tests enable the timely diagnosis of malaria even in remote, underserved areas and can be reliable and relatively low-cost, but are not quantitative and are difficult to quality control in the field. Other techniques, such as molecular diagnosis through polymerase chain reaction (PCR) are relatively costly in time and money, require considerable technical resources and/or are less suited to routine case management. Thus, light microscopy continues as the reference standard for diagnosing malaria disease in symptomatic people, and plays an important role in defining the infecting species, which also has impact on management of infections.

Light microscopy is a skills-based diagnostic procedure and accuracy depends on the microscopy technician’s competence, quality of the blood film, staining quality and the conditions of the microscope used. The reliability of field-based microscopy varies widely across and between countries, and poor standards can make diagnostic results irrelevant or even dangerous [5-11]. A general lack of internationally recognized competency standards has restricted the assessment and benchmarking of microscopists’ skills, restricting the ability of programmes to prioritize microscopy quality and advocate for support.

### Description of the external competency assessment (ECA) programme

#### Programme development

Based on evidence of highly variable microscopy standards during limited assessments in the Western-Pacific Region, and a perception of wider problems in microscopy competency, the WHO Regional Offices for the Western Pacific (WHO/WPRO) and Regional Office for South East Asia (WHO/SEARO) convened an international meeting in April 2005 in Kuala Lumpur, Malaysia. The meeting assessed the state of malaria microscopy and reviewed existing quality assurance (QA) systems, including those being developed at that time by WHO/WPRO, and identified models for the sustainable improvement of standards [12]. General principles were adopted for minimum standards necessary for a country to have a credible microscopy QA system:

Routine testing of microscopist competency through use of reference slide sets or slide bank;

Remedial training of microscopists who fail routine evaluation;

International accreditation of competency of microscopists at national reference level;

Cross-checking of slides submitted to reference or higher level microscopists in numbers that are sustainable and statistically relevant; and

Procurement and provision of quality microscopy supplies.

Following the 2005 meeting, WHO/WPRO and WHO/SEARO, in collaboration with partners including the Asian Collaborative Training Network for Malaria (ACTMalaria) and the Research Institute for Tropical Medicine (RITM, Philippines), commenced development of a bi-regional programme to assess and accredit malaria microscopy competency according to these recommendations, to standardize microscopy techniques and to help build the capacity of national reference laboratories to act as focal points for national QA programmes. This project, with support from the Australian Agency for International Development (AusAID) and the United States Agency for International Development (USAID), built on a slide bank and microscopy pre-qualification programme originally developed for the WHO programme to evaluate malaria RDTs by WHO/WPRO, the RITM and the University of the Philippines College of Public Health (UP-CPH). It incorporated further aspects of models developed for the Philippine national programme by UP-CPH and RITM, and elsewhere by Medicines Sans Frontiers (MSF) and the Kenyan Medical Research Institute (KEMRI) [13,14]. The constituents of the reference slide set and the competency standards were further refined at WHO expert consultations convened later in Geneva.

### Programme description

**Figure 1:**
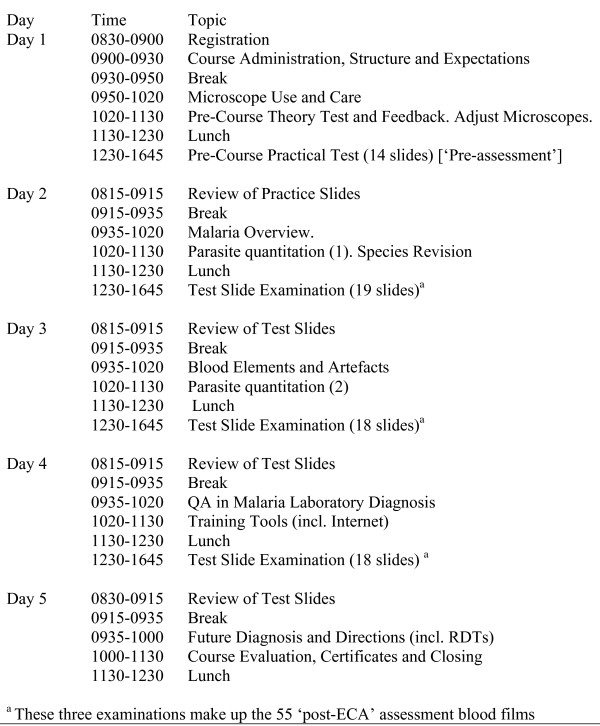
External competency assessment of national malaria microscopists: timetable.

The programme consists of competency assessments conducted in-country during a five-day skills update programme, using an external facilitator (see Figure 1). The national malaria programme selects the participants, requested to be experienced microscopists playing key roles in the national programme in a reference or supervisory role. The programme is coordinated by ACTMalaria.

By the end of 2011, 60 competency assessment and accreditation exercises (ECA) had been conducted in 14 countries in Asia; Australia, Bangladesh, Cambodia, China, Indonesia, the Lao PDR, Malaysia, Myanmar, the Philippines, Papua New Guinea, Solomon Islands, Thailand, Vanuatu, and Vietnam. A further ECA was conducted in Kenya during transfer of methods to the African Region, and more recently in Timor Leste. Each exercise was attended by approximately 12 microscopists. The competency level in pre-exercise assessments varied widely, reflecting a diversity of competency of high-level microscopists and current lack of pre-existing standardization in malaria microscopy programmes.

The ECA programme has a number of basic objectives geared towards the development of a successful programme, as agreed during the 2005 Kuala Lumpur meeting:

1. Development of a national reference (core) group of expert microscopists in participating countries, accredited to internationally recognized standards for malaria microscopy. This group should act as a catalyst and resource to improve microscopy standards, and form (ideally) a multi-country network of high-standard reference microscopists for external training and assessment of other countries within the network.

2. Formal certification of the competency of each microscopist, with potential to:

i. Provide recognition of the skill levels of individual microscopists, providing legitimacy within programmes for cross-checking and training;

ii. Standardize malaria microscopy practices between countries, facilitating standardization of monitoring activities and clinical studies;

iii. Raise self esteem and confidence, providing recognized and attainable goals;

iv. Enhance career development through linkage to a defined career structure.

Under coordination by ACTMalaria, participants are selected by the national programme, which is expected to support most in-country costs. WHO is involved through the regional offices and through its country offices in the decision-making process and in programme management, including confirmation of certificates of competence and assistance with national slide-bank development.

### Regional slide bank

A regional slide bank, housed at RITM in Manila and funded through the WHO, acts as the reference panel against which competence is assessed. This was developed based on the recommendations in the WHO microscopy QA manual [15]. Briefly, the bank contains slides from over 100 parasitaemic donors (predominantly *Plasmodium falciparum* and *Plasmodium vivax*, with other species in low numbers; seven *Plasmodium malariae* and *three Plasmodium knowlesi* by end 2011) from the Philippines and Cambodia, collected by the respective national programmes according to Standard Operating Procedures developed for the purpose. One hundred slides are included from each individual, including a thick film of 6 μL blood made against a 12 mm diameter template, and a thin film. Slides are stained with 3% Giemsa for 30 to 45 minutes and protected with a coverslip. In some cases, blood is diluted according to procedures established by the WHO-FIND malaria RDT evaluation programme to ensure availability of adequate low-parasite density slides [16]. Further parasite-negative slides are prepared from donor blood. The bank maintains a database tracking slide loans and availability.

Each patient donation was validated using simple nested polymerase chain reaction (PCR) and again in 2012 using real time PCR, by Institut Pasteur in Cambodia for the five malaria parasite species known to infect humans [17]. Six expert microscopists performed blinded microscopy readings on two slides each from each patient (drawn from a pool of expert microscopists, more recently accredited as Level 1 by this programme, and drawn from at least two countries.

### Structure of the assessment

While the five-day duration and the structure of the assessment exercise has remained generally consistent over the years of operation, certain changes are ongoing to improve the quality of the programme, particularly the quality of the slides and teaching materials. The ECA facilitator is a WHO-certified Level 1 (Expert) microscopist with wide experience in teaching and laboratory quality assurance, with further facilitators in training.

**Figure 2:**
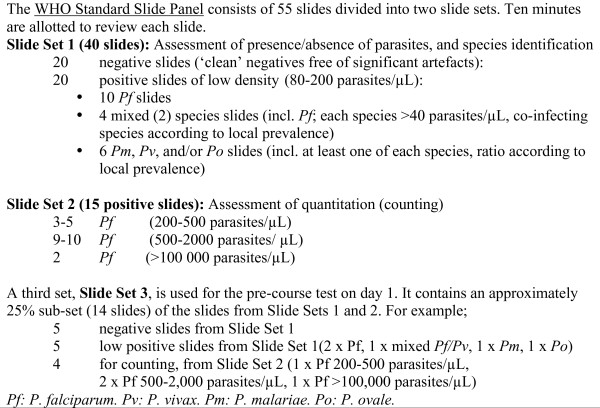
WHO Standard Slide Panel used for competency assessment.

The ECA structure (see Figure 1) utilizes initial assessments to provide a ‘baseline’ evaluation of each participant’s theoretical knowledge and practical skills, especially their proficiency in parasite species identification and parasite quantitation, followed by 55 slides examined over three days for the ‘post-ECA assessment’. Fourteen slides are examined for the pre-ECA practical assessment. The ‘post-assessment’ was originally on day 4 during the early iterations of the programme and consisted of 40 slides, but was then distributed through the course to reduce the stress of a nearly full day of assessment, and expand the number of slides that can be assessed. Interspersed audiovisual presentations review relevant aspects of microscopic diagnosis, including malaria parasite stage and species identification and the need to standardize techniques for parasite quantitation, and the post-assessment is structured to follow relevant update modules. The review sessions also focus on extensive feedback and address particular weaknesses identified during the slide examination sessions. Slides utilized for testing include *P. falciparum*, *P. vivax*, *P. malariae* and may include *Plasmodium ovale* (rare in Asia) if available, and include mixed infections and examples of varying parasite densities. Slides are provided by the WHO/WPRO Regional Slide Bank at RITM and described in Figure 2.

**Figure 3:**
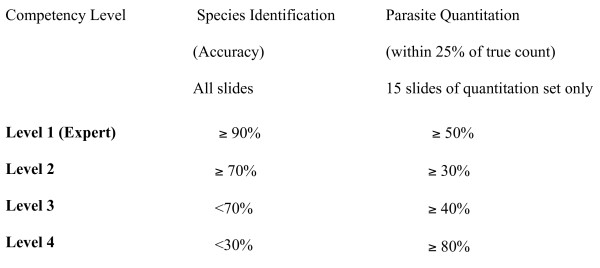
WHO competency levels for accreditation of malaria microscopists.

All participants are accredited with a competency score from 1 to 4 at the end of the ECA, based on the ‘post-ECA’ assessments (see Figure 3). The scores for species identification are calculated according to the number of correct species identified (two points per correct identification, one point for one species in mixed infections). For parasite quantitation, the score depends on the closeness to the true count (within 25% = one point). The final score includes both scores to represent the overall performance of the microscopists, and is reflected in a certificate presented on the final day, signed by the course facilitator, ACTMalaria coordinator, and WHO representative. The accreditation is considered by the programme to be a valid demonstration of competency for three years. Although it can be assumed that there is a malaria diagnostics quality assurance programme within the country, as specified by the WHO QA manual, for the trained reference level microscopists to transfer and monitor malaria diagnostics, it has to be acknowledged that often this is not the case. Either way this programme can be utilized to supplement a sustainable and reasonable QA programme when implemented.

This ECA exercise is too short to include any formal training in instruction and communications skills, or in the management of quality assurance programmes. It is recognized and emphasized that these are also essential for any national quality assurance programme but should usually be addressed more formally by those responsible for training. The competency levels and the accuracy required in species identification and parasite quantitation is detailed in Table 1, including species recognition and accuracy of parasite quantitation. Accreditation certificates are provided to each participant and are valid for three years before requiring formal re-confirmation.

**Table 1:** Results of mean pre- and post-ECA activity scores of WHO-ACTMalaria Malaria Microscopy ECAs from 2009-2010

**Countries**	**Number of Participants (n)**	**Species ID Mean Score**		**Parasite quantitation Mean Score**		**Difference in score means**	
		**Pre**	**Post**	**Pre**	**Post**	**Spec.ID Score Change, (p-)**	**Parasite quantitation Score Change, (p-)**
Country 1	5	55	62	20	38	7 (0.22)	18 (0.01)
Country 2	12	47	84	25	49	37 (0.002)	24 (0.14)
Country 3	9	72	84	28	43	12 (0.11)	15 (0.22)
Country 4	11	71	89	27	48	18 (0.008)	21 (0.05)
Country 5	12	88	90	25	50	2 (0.64)	25 (0.001)
Country 6	12	68	89	46	51	21 (<0.001)	5 (0.56)
Country 7	12	68	84	21	38	16(0.004)	17 (0.03)
Country 8	12	38	83	24	38	45 (0.61)	14 (0.08)
Country 9	23 (in 2 sessions)	91	96	30	58	5 (0.003)	28 (<0.001)
Country 10	11	65	75	13	31	10 (0.001)	18 (0.05)
Country 11	12	60	74	15	39	14 (0.01)	24 (0.18)
Country 12	12	75	87	27	54	12 (<0.001)	27(0.001)
Country 13	8	31	66	3	21	35 (0.06)	18 (0.04)
Country 14	12	82	93	46	47	11 (0.001)	1 (0.93)

p≤0.05 indicates significant difference between pre- and post ECA assessments (matched pairs t-test).

## Methods

Quantitative analysis of the competency scores obtained in the ECA programme were based on the most recent ECA from each of the 14 countries participating in the programme, including 424 microscopists up to the end of 2010. Overall mean scores for species identification and parasite quantitation are calculated for each country, together with the range and average of mean scores between countries. The pre- and post-assessment score means were compared using paired sample *t*-tests to determine whether there was a significant change in their scores due to the retraining session (Table 1) using SPSS 14.0.

The impact of, and attitudes to, the ECA programme were formally evaluated through two reviews conducted in 2008 and 2010. The objective in each case was to determine the usefulness of the accreditation course for participating national malaria programmes (NMPs) and to describe areas where improvement in design would be of benefit.

Both reviews aimed to:

Assess the programme with regard to its content and usefulness to participants and NMPs;

Outline QA programmes in target countries and assess the ECA’s ‘fit’ with the existing framework; and

Make recommendations to NMPs on how to increase the utility and effectiveness of ECA to improve microscopy technicians’ diagnostic abilities through the analysis of pre- and post-ECA performance by participants, and through interviews with past participants, key persons involved in the development of the programme, and people involved in malaria programmes of countries where evaluations had been conducted.

Using the course facilitator’s ECA reports from 2008–2010, a matrix was created to highlight the impact and effectiveness of ECA conducted in collaborating countries. The matrix also assessed national malaria laboratories for quality assurance programmes and activities such as the routine cross-checking of slides, regular training and retraining courses and supervisory visits.

Surveys were designed to assess the activity of national QA programmes; including crosschecking (validation), retraining courses and evidence of the regular utilization of ECA participants. Questionnaires were sent by email to past participants (microscopists), NMP managers and WHO country office malaria officers and in-depth interviews conducted with individuals ranging from different levels of malaria QA programmes, ranging from ECA facilitator to national level microscopists. The advantages and weaknesses of the ECA system were discussed, highlighting the effectiveness of the review/revision component in improving diagnostic performance and the ability of the ECA facilitator to address and clarify participants’ questions, and identified deficiencies. Discussions also included the improvements in microscopist competencies, feedback delivery mechanism of ECA reports, quality of regional slide bank, training equipment, and facilitator-student interaction.

## Discussion

### Changes in skills and competency levels of ECA participating microscopists

According to analysis of pre- and post-ECA accreditation scores, microscopists from five countries showed significant changes in their scores in both species identification and parasite quantitation (Table 1).

### Species identification

**Figure 4:**
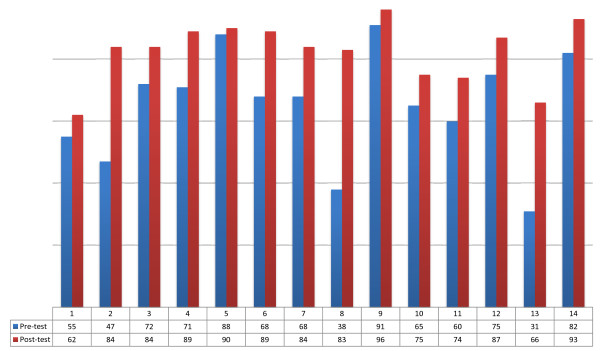
Differences in mean species identification scores among participants from 14 countries, 2009–2010.

Comparison of the means of pre- and post-assessment scores: all countries showed marked improvement in species identification, with a mean increase of 17.5 points (27% improvement on initial mean score) -- ranging from seven to 45 (Table 1 and Figure 4).

### Quantitation

**Figure 5:**
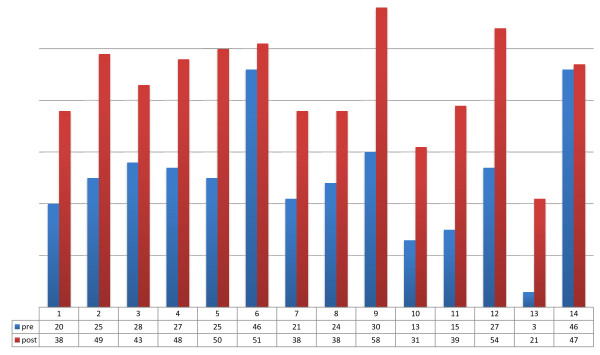
Differences in mean malaria parasite quantitation scores among participants from 14 countries, 2009–2010.

Parasite counting improved markedly in all countries from start to completion of the ECA courses, with readings obtaining counts within 25% of the ‘true reading’ increasing by an average of 18 percentage points, ranging from one to 28 points (a 72% improvement on initial score) (Table 1 and Figure 5).

Overall, 82% of readings correctly identified parasites in post-ECA testing, compared to 42% of readings providing accurate counts (within 25% of the ‘true reading’) for parasite quantitation.

### Interviews

Key informant interviews focused on specific areas depending on the perceived knowledge, experience and expertise of each informant; however, opinions and information gained from interviews reiterated similar themes including mechanisms and the public nature of ECA report delivery. One recurring issue raised was the need to improve the quality of the reference slides used and the recommendation for the preparation of new slides by the regional slide bank. Both are difficult issues. Results are provided publically to emphasize transparency and the importance of using competency as a basis for validation and expertise within programmes. Regarding slide quality, the slides are made by experienced technicians, subject to quality control and well-validated, but some variation in quality is inevitable when producing large slide sets. However, although competency includes the ability to interpret imperfectly prepared slides, consistency and quality across the ECA slide set are far greater than encountered in the field and should therefore constitute a fair assessment.

Many participants had concerns regarding the assessment’s emphasis on application of the new parasite quantification method recommended in the WHO QA manual [15]. The method followed by the ECA programme caused some confusion among participants used to alternative methods; several participating countries continue to use the “plus method” and are unfamiliar with more accurate methods. Very few record their results as parasites/μL during routine work. The emphasis on quantitation methods during the course probably accounts for the improvement by most participants in the post-ECA assessment. Parasite quantitation clearly needs to be a continued focus of additional training as quantitation is one of the main advantages of light microscopy, and dissemination of correct counting methods to other microscopists in countries should be emphasized [10,15]. It is unusual for a major disease programme such as malaria to have had such variation in parasite quantification methods in the past.

Participant feedback also revealed particular difficulties faced by some microscopists with identification of some parasite species, particularly *P. ovale* and *P. malariae,* and this was sometimes considered to unfairly impact on microscopists from areas where these two species are rare. However, as the programme aims to test the inherent expertise of national reference-level microscopists, it can be safely argued that microscopists at this level should have the skills to diagnose less commonly seen malaria species and unusual cases.

### Implications of the ECA for participants and national programmes

The ECA programme in the Asia Pacific has demonstrated that marked improvement in the competency of experienced microscopists can be obtained between pre- and post-ECA exercises after only three to four days of consolidated revision and reviewing techniques. The improvement of 27% in species identification illustrates this rapid impact of correction of basic errors. Run currently on a budget of approximately 12,000–17,000 USD per course per country (including costs of an international facilitator), excluding the cost of the maintenance and replenishment of the Regional Malaria Slide Bank at RITM, it demonstrates that large inroads can be made in improving capacity without high resources. ECA costs are predominantly absorbed within country budgets, but designated central funding is required to maintain the bank and coordination.

The function of the accreditation programme is dependent on the adequacy of the regional slide bank described earlier, and its success is dependent on accuracy of the specified results of each slide. This was achieved through development and use of standard SOPs for slide preparation and emphasis on accuracy of validation: it included six pre-qualified microscopists and use of PCR as final arbiter of species. This is necessary, as it is essential that participants are not penalized for reporting species that are at very low density and missed by validators through chance. Slide production, validation and filing required considerable personnel time and a dedicated staff, and a budget for ongoing bank maintenance. The bank collection was undertaken by Ministry of Health staff, and the respective programmes involved retained 50% of slides for national use, which enabled national programmes to dedicate personnel time to the project. However, the funding for the bank, while modest, has been difficult to secure as it does not fit well with the bilateral funding model followed by most donors in this field. An enlightened and flexible approach by USAID/Asia staff enabled this project to continue. Dedicating funds for such ‘common good’ projects would be a low-cost, high-impact improvement to some funding budgets.

Introducing an accreditation of competency can be highly threatening to members of any profession, who are unfamiliar with it. It opens the unwanted possibility of revealing skills-based weaknesses before colleagues and supervisors, possibly with potential career and financial implications. Accreditation can also highlight, by implication of non-participation, a lack of technical competence of senior officials and managers who do not actively participate in the ECA. Data collected from ECA participant feedback forms, distributed surveys, and key informant interviews suggest that the majority of participants view the ECA programme positively, despite the possible risk to reputation arising from the policy of making competency results available to the programme and colleagues. In addition to improving existing abilities to identify species and quantify parasites, ECA activities are an opportunity to update wider knowledge and microscopy skills. As participants showed considerable improvement between pre- and post-assessment, the accreditation exercise should help to build significant levels of self-confidence in skills and knowledge. In turn, it is hoped this will lead to increased respect for, and trust in, their competency by supervisors, colleagues whose slides they are crosschecking, and among clinicians who need to have trust in diagnostic results.

The value of ECAs will be greatly enhanced if the results are disseminated back to the national malaria programmes and result in an early impact on the programmes. In order to maximize effectiveness and sustainability of ECA activities, it is imperative that continued refresher training and crosschecking by the competent participants be integrated into national malaria programmes. However, key informants frequently noted that an adequate mechanism was lacking to share recommendations from the ECA facilitator through the WHO to countries concerned and to then follow them up. Currently, the course facilitator leaves a draft of the hard copy of the ECA report with the host laboratory and/or programme manager as well as the WHO country representative; a final copy of the report is sent to the WHO Regional Office as well as the coordinator, ACTMalaria. Although the course facilitator is able to briefly discuss findings and recommendations before leaving a country, it is apparent from the results that some programmes are unable to make effective use of the findings. Consequently, pro-active measures are being taken by WHO to enhance the effective communication of course recommendations. WHO country representatives and malaria personnel now facilitate the dissemination of findings and recommendations to national programmes, in collaboration with ACTMalaria. A recent WHO-organized regional malaria programme managers meeting focused on quality assurance for malaria diagnosis, informed by this needs assessment, to initiate a revision of country malaria QA systems work plan.

WHO gives clear recommendations for a hierarchical structure for national malaria microscopy QA programmes, based on regular competency assessments and retraining within the national system, supported by a national slide bank [15]. However, banks of well-prepared, stained examples of malaria parasites are uncommon. Parasite specimen collection and the validation of slides, including PCR, require significant resources and skills often lacking within national programmes. National slide banks must have credibility regarding the validity and accuracy of its slides, sometimes meaning external validators are required. While the WHO manual recommends a simplified crosschecking system, this still requires significant logistical organization and availability of technical expertise. While the ECA seeks to put essential elements of this in place, it appears most countries have not fully utilized this, either through lack of resources or through giving it insufficient priority.

The increase in skills within and between courses, and the potential of the programme to catalyze skills development, is demonstrated well in the Philippines, where seven ECAs have been held and a national structure is in place to utilize reference microscopists and accredit all microscopy technicians within the national system. In three ECA activities conducted in the Philippines, microscopists averaged 94%, 91%, and 91% accuracy respectively in the pre-ECA assessments on parasite species identification. Despite having little room to improve, these microscopists further improved their ability to identify different species of malaria parasites (averaging 95%, 96% and 96% accuracy in post-ECA assessments). High performance on pre-ECA assessments is a good indicator that microscopists regularly participate in ongoing training and maintain high levels of competence. All of the Philippine microscopists assessed during the three recent ECAs achieved Level 1/Expert or Level 2 accreditation, and these reference microscopists are active in slide validation/crosschecking and supervisory visits. Additionally, regular ongoing training for microscopists is in place within the national programme with emphasis on weak areas such as parasite quantitation.

### Further limitations of current ECA programme

The contents of the Regional Malaria Slide Bank, and consequently the slide sets used in the assessments, varied somewhat across time as lost slides were replaced and the bank expanded, but the evaluation set remained within the parameters in Figure 2. There will inevitably be variation in ease of parasite recognition between slides from different patients, and in accuracy of quantitation of individual slides, which are based on averages of 12 expert readings from slides from the same set. However, the ability of reference microscopy cadres from some countries to achieve high frequency of Level 1 results after retraining, uniformly in ECA in the Philippines, indicates that, despite some variability in slide quality, a microscopist with sufficiently high expertise can expect to have that competency reflected in a Level 1 rating.

Recruitment of suitable facilitators was a chronic difficulty encountered in this programme. A facilitator must be a highly competent microscopist with a WHO Level 1 accreditation, a positive attitude and proven instructional and facilitation skills, as well as the time to dedicate to the task. Collaboration between Regions is needed to provide such a pool of facilitators for the ECA exercises, providing more sustainability to the programme. Synergies may also be possible with microscopy QA programmes for other diseases, such as tuberculosis.

## Conclusions

The ECA programme establishes and improves microscopy technicians’ diagnostic skills and promotes internationally standardized microscopy techniques, with particular emphasis on areas of weakness. Despite the short duration of each ECA, participants have demonstrated significant improvements in their abilities to identify malaria species and to accurately quantify parasites. While some areas of the ECA exercises require further development and modification, the system works and has proven to be implementable on a wide scale. Transfer of methods has already occurred to the African Region, where the African Medical Research and Educational Foundation (AMREF) coordinates assessments with the WHO Regional Office for Africa, based on the same standards. The ECA model can already be expanded to other Regions of WHO to improve malaria microscopy diagnosis and consequently, national and international malaria control and elimination efforts. This will require a consistent level of support, but will provide much needed international standardization and predictability, both for research and for malaria case management.

## Abbreviations

ACTMalaria: Asian Collaborative Training Network for Malaria; AMREF: African Medical Research and Educational Foundation; AusAID: Australian Agency for International Development; ECA: External competency assessment; FIND: Foundation for Innovative New Diagnostics; KEMRI: Kenyan Medical Research Institute; MSF: Médecins Sans Frontières; PCR: Polymerase chain reaction; QA: Quality Assurance; DT: Rapid diagnostic test; RITM: Research Institute for Tropical Medicine; UP-CPH: University of the Philippines College of Public Health; USAID: United States Agency for International Development; WPRO: WHO Regional Offices for the Western Pacific; WHO/SEARO: WHO Regional Office for South East Asia; WHO: World Health Organization.

## Competing interests

The authors declare they have no competing interests.

## Authors’ contributions

AK and SA jointly led the evaluation of the programme, analysis and the writing of this paper. BF and DB coordinated the review process. KL acted as the key facilitator/trainer and technical expert from the commencement of the international assessment process, and provided the reports upon which much of the data here is based. DB, KL, EMC, CH, JL jointly conceived and led programme development, JL, CH, EMC and DB led development of the regional slide bank. All authors reviewed and revised the manuscript. All authors read and approved the final manuscript.
